# C-857-03. Burn Survivor Agreement with Established Philosophical Models of Disability

**DOI:** 10.1093/jbcr/irag033.103

**Published:** 2026-04-06

**Authors:** Karel-Bart Celie, Kushal Paul, Eloise W Stanton, Cindy Rutter, Maxwell B Johnson, T Justin Gillenwater, Haig A Yenikomshian

**Affiliations:** Division of Plastic and Reconstructive Surgery, Keck School of Medicine, University of Southern California, Los Angeles, CA; University of Southern California, Los Angeles, CA; Division of Plastic and Reconstructive Surgery, Keck School of Medicine, University of Southern California, Los Angeles, CA; 1 Amazing Life, Carlsbad, CA; Division of Plastic and Reconstructive Surgery, Keck School of Medicine, University of Southern California, Los Angeles, CA; Division of Plastic and Reconstructive Surgery, Keck School of Medicine, University of Southern California, Los Angeles, CA; Division of Plastic and Reconstructive Surgery, Keck School of Medicine, University of Southern California, Los Angeles, CA

## Abstract

**Introduction:**

Three philosophical models of disability that have been robustly defended in the ethics literature are the medical model, the social model, and—most recently—the welfarist model (Table 1). Each model has different implications for policy and practice. No empirical ethics study to date has specifically interrogated the degree to which burn survivors agree with the central “thesis” of each philosophical model.

**Methods:**

The study protocol was approved by our IRB (HS-23-00757). A survey describing the key thesis of each model of disability was administered in person to adult burn survivors at an outpatient clinic for a large regional burn center between July and September of 2025. Descriptive statistics were calculated, and proportions of agreement and disagreement were compared across models using chi-square analysis (alpha = 0.05).

**Results:**

Fifty-six burn survivors completed the survey (mean age 38.8 ± 15.4 years; mean %TBSA 21.2 ± 22.8). Twenty-two (39.3%) burn survivors self-identified as having a disability. Among the three models of disability, the central thesis of the welfarist model drew the highest proportion of strong agreement (21.4%) compared with the medical (14.3%) and social (10.7%) models (Fig. 1). The overall proportion of agreement across models was not significantly different (*p*=.39). However, in response to a forced-choice question, 64.3% (36/56) selected the welfarist model, 25.0% (14/56) the social model, and 10.7% (6/56) the medical model. Nearly all respondents (98.2%, 54/55) characterized disability as instrumentally—rather than intrinsically—undesirable; this corroborates a core feature of the welfarist model of disability. Burn survivors who self-identified as having a disability showed significantly stronger agreement with the welfarist model compared to those who did not self-identify as disabled (*p*=.011).

**Conclusion:**

Burn survivors at a large regional burn center were most likely to strongly endorse the central thesis of the welfarist model of disability. This tendency was particularly strong when respondents self-identified as having a disability.

**Applicability to Practice:**

This is the first time burn survivors have been directly queried regarding the central thesis of each of the three philosophical models of disability. Our findings suggest a need to re-examine conventional accounts of disability used in policy, research, and clinical care.

